# The diet supplemented with raw and hydrolyzed *Microchloropsis gaditana* and *Arthrospira platensis* blend modulates intestinal microbiota and metabolomic profiles in *Sparus aurata*

**DOI:** 10.1007/s10695-026-01791-0

**Published:** 2026-09-14

**Authors:** Sonia Rohra-Benítez, Jorge García-Márquez, Rocío Bautista-Moreno, Fernando Vallejo, María Isabel Sáez-Casado, Juan Antonio Martos-Sitcha, Miguel Ángel Moriñigo, Silvana Teresa Tapia-Paniagua

**Affiliations:** 1 Department of Microbiology, Faculty of Sciences, CEI·MAR-International Campus of Excellence in Marine Science, University of Malaga, Málaga, Spain; 2 Bioinformatic Unit of – Super Computer and Bioinnovation Center (SCBI), University of Malaga, Málaga, Spain; 3 Research Group On Quality, Safety, and Bioactivity of Plant Foods, Department of Food Science and Technology, CEBAS-CSIC, 30100 Murcia, Spain; 4 Department of Biology and Geology, CEI·MAR-International Campus of Excellence in Marine Science, University of Almería, Almería, Spain; 5 Department of Biology, Faculty of Marine and Environmental Sciences, Instituto Universitario de Investigación Marina (INMAR), CEI·MAR-International Campus of Excellence in Marine Science, University of Cádiz, Cádiz, Spain

**Keywords:** *Arthrospira platensis*, *Microchloropsis gaditana*, Aquafeed, Intestinal microbiota, Metabolomics, *Sparus aurata*

## Abstract

The transition toward sustainable aquaculture has prompted the search for alternative feed ingredients that support fish health while reducing dependence on fishmeal. Microalgae such as *Microchloropsis gaditana* and *Arthrospira platensis* are rich in bioactive compounds with potential functional benefits. This study evaluated the effects of supplementing gilthead seabream (*Sparus aurata*) diets with a 10% blend of raw or enzymatically hydrolyzed *M. gaditana* and *A. platensis* on intestinal microbiota composition and metabolomic profiles. Over a 41-day feeding trial, fish were fed either a control diet or diets supplemented with raw or hydrolyzed microalgae. Microbial communities were assessed via 16S rRNA gene sequencing, and intestinal metabolites were profiled using UPLC-QTOF and GC–MS. The hydrolyzed microalgae diet induced a marked shift in microbial composition, characterized by a significant increase in Proteobacteria and reduced alpha diversity, particularly in the anterior intestine, whereas the raw diet preserved microbial diversity and promoted the presence of beneficial taxa such as *Fusobacteria*. Both supplemented diets led to a reduction in inflammation-related metabolites, including 12-keto-leukotriene B4 and 15-keto-prostaglandin E2, and facilitated the accumulation of beneficial long-chain fatty acids, such as oleic and stearic acids. Overall, these findings indicate that dietary inclusion of *M. gaditana* and *A. platensis*, particularly in hydrolyzed form, can modulate gut microbiota and intestinal metabolic responses in *S. aurata*, supporting their potential use as functional and sustainable aquafeed ingredients.

**Supplementary Information:**

The online version contains supplementary material available at https://doi.org/10.1007/s10695-026-01791-0.

## Introduction

The growing demand for sustainable aquaculture practices has accelerated the search for alternative dietary ingredients that can reduce reliance on fishmeal without compromising fish health and performance. Fishmeal, long considered a cornerstone of aquafeeds due to its high protein and lipid content, is increasingly associated with environmental concerns such as overfishing and phosphorus pollution, as well as economic instability (Sampath et al. 2020; Liang et al. 2022; Hussain et al. 2024). As a result, identifying novel, nutritionally valuable, and cost-effective ingredients has become a priority in aquaculture research (Cerezo-Ortega et al. 2021).

Microalgae have emerged as promising candidates in this context, offering high protein content, essential fatty acids, and a range of bioactive compounds with potential health-promoting effects (Nagappan et al. 2021; Idenyi et al. 2022; Trevi et al. 2023). Among them, *Microchloropsis gaditana* (Chlorophyta) is notable for its rich content of omega-3 polyunsaturated fatty acids (PUFAs), including eicosapentaenoic acid (EPA) and docosahexaenoic acid (DHA), as well as antioxidants that enhance fish immunity, growth, and metabolic function (Otero et al. 1997; Ayala et al. 2020; Lozano-Muñoz et al. 2020; Cerezo-Ortega et al. 2021). Studies have demonstrated that dietary inclusion of *M. gaditana* increases EPA and vitamin D3 levels in fish tissues and positively modulates intestinal health (Lozano-Muñoz et al. 2020; Sáez et al. 2022).

Similarly, the cyanobacterium *Arthrospira platensis* (commonly known as Spirulina) offers high nutritional value due to its content of protein, essential amino acids, fatty acids, vitamins, minerals, and other bioactives (Niccolai et al. 2019; Galafat et al. 2022; Peralta-Sánchez et al. 2024). Its inclusion in aquafeeds has produced beneficial outcomes across several fish species, such as Nile tilapia (*Oreochromis niloticus*) (Mahmoud et al. 2018), yellow catfish (*Pelteobagrus fulvidraco*) (Liu et al. 2019), and gilthead seabream (*Sparus aurata*) (Galafat et al. 2022), improving gut function, oxidative stress resistance, and immune status. Notably, *A. platensis* has minimal effects on overall microbiota diversity (Peralta-Sánchez et al. 2024), which may be advantageous in preserving gut microbial balance.

Given the complementary nutritional and functional properties of *M. gaditana* and *A. platensis*, their combined inclusion in aquafeeds represents a promising strategy to develop balanced functional additives. While *Arthrospira* provides highly digestible protein and bioactive compounds with immunomodulatory potential, *Nannochloropsis* is particularly rich in EPA, pigments, and antioxidant molecules. Therefore, their combination may result in additive or synergistic effects on intestinal microbiota and host metabolism, while reflecting the formulation strategies currently adopted in commercial aquafeeds.

Despite these benefits, the bioavailability of microalgal nutrients is often constrained by structural barriers, particularly the cellulose-rich cell wall of *M. gaditana*, which limits the release and absorption of intracellular compounds (Ayala et al. 2020). Enzymatic hydrolysis has been proposed as an effective strategy to improve nutrient accessibility by degrading cell wall structures and releasing bioactive peptides and free amino acids with potential functional effects (Bongiorno et al. 2022).

Beyond their nutritional contributions, microalgae may also shape the gut microbiota, a key player in fish digestion, immune modulation, and overall metabolism (Cerezo-Ortega et al. 2021). In *S. aurata* juveniles, hydrolyzed *A. platensis* has been shown to enhance intestinal mucosal integrity, boost enzymatic activity, and reduce lipid peroxidation (Galafat et al. 2020). Similarly, dietary *M. gaditana* has been linked to modifications in gut microbiota composition and elevated levels of bioactive metabolites such as PUFAs and polyphenols, which may underlie reduced inflammation and improved gut function (Sharma et al. 2019; García-Márquez et al. 2022; Mueller et al. 2023; Sáez et al. 2022). Moreover, hydrolyzed *M. gaditana* has been associated with changes in fish physiology and muscle quality, even in the absence of significant growth effects (Sáez et al. 2022).

Despite these promising results, most prior studies have focused primarily on zootechnical parameters, histology, or physiological endpoints, with limited emphasis on the interplay between microalgal processing, gut microbiota, and host metabolism (Ayala et al. 2020). A more integrated approach is needed to elucidate how raw versus hydrolyzed microalgae affects intestinal microbial communities and metabolomic output.

The present study aims to assess the impact of supplementing finishing diets for *S. aurata* with a 10% blend of raw or enzymatically hydrolyzed *M. gaditana* and *A. platensis* on gut microbiota composition and intestinal metabolomic profiles. Using 16S rRNA gene sequencing and untargeted metabolomics (UPLC-QTOF and GC–MS), this work seeks to uncover the effects of microalgal processing on microbial ecology and host metabolic responses, with the goal of supporting the development of functional, sustainable aquafeeds.

The present study builds upon a previously published feeding trial (Molina-Roque et al. 2022), which reported significant improvements in growth performance, feed efficiency, stress-related markers, and intestinal functional parameters in fish fed the same experimental diets.

## Material and methods

### Experimental diets

Three iso-nitrogenous (42.5% crude protein on a dry weight basis, DW) and iso-lipidic (17.3% crude lipid, DW) experimental diets were formulated. Two diets contained 100 g kg⁻^1^ of microalgae-based additives: LB-ChromaBream (“raw”) and LB-ChromaBream-plus (“hydrolized”), while a third additive-free diet was used as the control (Supplementary Table S1). The additives were supplied by LifeBioencapsulation S.L. (Almería, Spain) as freeze-dried concentrated products composed of a blend of two microalgae species (70% *A.* *platensis* and 30% *N.* *gaditana*). The algal biomass was incorporated either in raw form or after enzymatic hydrolysis. The hydrolysis process was carried out using a combination of commercial proteases under controlled conditions, as described by Galafat et al. (2022) (Supplementary Table S1).

The experimental diets were manufactured at the CEIA3–Universidad de Almería facilities (Servicio de Piensos Experimentales, http://www.ual.es/stecnicos_spe; Almería, Spain) following standard aquafeed processing procedures. Briefly, feed ingredients and algal biomass were thoroughly mixed, and water was added (up to 300 g kg⁻^1^) to obtain a homogeneous dough using a vertical helix ribbon mixer (Sammic BM-10, Sammic, Azpeitia, Spain). The dough was then processed through a single-screw laboratory extruder (Miltenz 51SP, JSConwell Ltd., New Zealand) to produce pellets with a diameter of 5 mm. The extruder barrel consisted of four sections with a temperature profile (from inlet to outlet) of 90, 92, 95, and 105 °C. The pellets were subsequently dried at 30 °C for 24 h in a 12 m^3^ forced-air drying chamber (Airfrio, Almería, Spain) and stored at − 20 °C until use.

The formulation and proximate chemical composition of these experimental diets have been previously described in detail in Sáez et al. (2024), where the same dietary formulations were used. For clarity and accessibility, a summary of the ingredient composition, proximate analysis, and fatty acid profiles of the three experimental diets is provided in the Supplementary Material (Supplementary Tables S1–S2).

### Feed preparation

The experimental diets for gilthead seabream (*Sparus aurata*) were developed at the CEIA3–University of Almería facilities (Servicio de Piensos Experimentales, http://www.ual.es/stecnicos_spe). The control diet followed a standard commercial formulation, as outlined by Molina-Roque et al. (2022). Building upon this base, two additional experimental diets were formulated by incorporating 10% of commercial microalgae-based products. These two variants, referred to as the “raw” and “hydrolyzed” diets, included supplements supplied by LifeBioencapsulation S.L. (Almería, Spain). The supplements consisted of freeze-dried, concentrated biomass containing a blend of *Arthrospira platensis* (70%) and *Microchloropsis gaditana* (30%). The hydrolyzed version underwent enzymatic treatment with a mixture of commercial proteases under controlled conditions, following the methodology described by Galafat et al. (2022). All ingredients, including the microalgae supplements, were thoroughly mixed with water (up to 300 g/kg) to achieve a uniform dough. This mixture was processed using a vertical helix ribbon mixer (Sammic BM-10, Sammic, Azpeitia, Spain) and subsequently extruded through a twin-screw laboratory extruder (Evolum 25, Clextral, France) to produce pellets with a diameter of 5 mm.

### Experiment design and sampling

Prior to the start of the feeding trial, all fish were fed the control diet for a 15-day acclimatization period to standardize baseline physiological and intestinal conditions across treatments. Three experimental diets were evaluated over a 41-day feeding trial using 108 gilthead seabream (*Sparus aurata*) juveniles with an initial mean body weight of 182.5 ± 1.8 g, following the general procedures described by Molina-Roque et al. (2022). Fish were individually weighed and randomly allocated into nine 400 L tanks (12 fish per tank; initial stocking density 5.47 ± 0.06 kg/m^3^) at the facilities of the Central Research Service in Marine Farming (SCI-CM), University of Cádiz. The trial was conducted from May 28th to July 8th, 2020, under an open water-recirculation system.

Fish were reared in an open-flow seawater system under controlled conditions, as previously described by Molina-Roque et al. (2022). Water temperature was maintained at approximately 19 °C, salinity at 36 ppt, and fish were exposed to the natural photoperiod of the region. Dissolved oxygen levels were kept within optimal ranges for gilthead seabream by continuous aeration. Fish were fed once daily to apparent satiety (ad libitum), ensuring homogeneous feeding conditions among experimental tanks.

Prior to the feeding trial, fish were acclimated to the experimental conditions for 10 days. Thereafter, the experimental diets were offered once daily at 10:30 h to apparent satiation (ad libitum), ensuring that all feed provided was consumed in each tank. To avoid bias, the three aquafeeds were coded with different colors without revealing their composition, thereby implementing a blind feeding design. No mortality occurred during the entire experimental period.

At the conclusion of the 41-day feeding trial, a final sampling was conducted. Fish were fasted overnight (approximately 16 h) prior to sampling to minimize short-term postprandial variability and reduce the presence of undigested feed residues in the intestinal lumen. Eighteen fish per dietary treatment (six per tank), previously fasted for 24 h, were randomly selected and subsequently measured and weighed. The euthanasia was performed using 2-phenoxyethanol at a concentration of 1 mL·L⁻^1^ (v/v) in seawater. Fish were euthanized by cervical section, and tissue biopsies were collected. For each sampled fish, both the anterior and posterior intestinal sections were collected and analyzed separately, as these intestinal compartments differ in their physiological function and microbial composition.

Intestinal samples were immediately frozen in liquid nitrogen and stored at − 80 °C until further analyses.

All experimental procedures adhered to the guidelines of the Ethics and Animal Welfare Committee of the University of Cádiz, in accordance with Spanish legislation (RD 53/2013 and RD 118/2021) and the European Directive (2010/63/EU), and were approved by the Ethical Committee of the Andalusian Regional Government (approval no. 16/16/2019/170).

Growth performance parameters (SGR, FE), hepatosomatic index (HSI), and cortisol levels from the same experimental fish were previously reported (Molina-Roque et al., year). A summary of these data is provided in Supplementary Table S3 for reference.

### DNA extraction and sequencing by Illumina Miseq technology

Intestinal samples of *S. aurata* (anterior and posterior sections) previously stored at − 80 °C were thawed gradually on ice. Intestinal contents were obtained by gently pressing the sections toward their ends with a sterile instrument. Genomic DNA was extracted from each sample following the saline precipitation protocol described by Martínez et al. (1998), with slight modifications introduced by Tapia-Paniagua et al. (2010). DNA yield and quality were assessed fluorometrically using the Qubit™ dsDNA HS Assay Kit (Thermo Fisher Scientific, Waltham, MA, USA), as well as by spectrophotometric and electrophoretic analyses to evaluate purity, quality, and integrity. Extracted DNA was stored at − 20 °C until further processing, and 30 ng of DNA was used for downstream analyses.

Amplification of the bacterial 16S rRNA gene was performed and sequenced on the Illumina MiSeq platform (Illumina, San Diego, CA, USA) at the Novogene Europe sequencing facility (Cambridge, UK), using a 2 × 250 bp paired-end strategy. The V3–V4 hypervariable regions of the 16S rRNA gene were targeted using primers 341 F and 806R.

Raw reads were processed for quality control: primers were trimmed with cutadapt, and read quality was assessed using FastQC (Andrews et al. 2010). Barcoded sequences were analyzed with the DADA2 pipeline in R (Callahan et al. 2016). Forward and reverse reads were truncated based on decreasing quality scores while ensuring sufficient overlap for merging. After error modeling and correction, paired-end reads were assembled into amplicon sequence variants (ASVs). Chimeric ASVs were identified and removed by comparison with higher-abundance parent sequences. Taxonomic assignment of representative ASVs was performed using the SILVA database (release 138), clustered at 99% identity and trimmed to the amplified region.

Microbial community composition and abundance were analyzed in R using the phyloseq package (McMurdie and Holmes 2013) and the vegan library (Oksanen 2017). Alpha diversity (Shannon index) and beta diversity (PCoA) were calculated. Statistical differences in diversity indices were evaluated; first normality of the data was evaluated using the Shapiro–Wilk test for each group. As deviations from normality were detected in some variables and the sample size was limited, non-parametric Kruskal–Wallis tests were applied for all group comparison (*p* < 0.05). For taxonomic analyses, ASVs with fewer than 10 reads in at least 10% of samples were filtered out. Taxonomic composition and abundance were visualized with the ggplot2 package (Alonso Cifuentes and González 2012). Differential taxonomic abundance was determined using DESeq2 (Love et al. 2014), applying a false discovery rate threshold of FDR < 0.05.

### Metabolomic analysis

#### Untargeted metabolomics by UPLC-QTOF

A total of six intestinal samples per dietary treatment (*n* = 18) were subjected to non-targeted metabolomic profiling following the protocol described by Barber et al. (2021). Both raw and hydrolyzed samples of gilthead seabream were analyzed using an ultra-performance liquid chromatography coupled to quadrupole time-of-flight mass spectrometry (UPLC-QTOF), operated in both positive and negative ionization modes.

For extraction, 150 mg of each sample was homogenized with 1 mL methanol, vortexed for 5 min, and centrifuged at 10,000 rpm for 10 min at 4 °C. The supernatant was filtered through a 0.22-μm membrane, transferred to glass vials, and 2 μL was injected into the chromatographic system. Analyses were performed on an Agilent 1290 Infinity LC system coupled to a 6550 I-Funnel Accurate-Mass QTOF (Agilent Technologies, Waldbronn, Germany) equipped with a dual electrospray ionization source (ESI-Jet Stream). Samples were separated on a Poroshell 120 EC-C18 column (3 × 100 mm, 2.7 µm; Agilent) at 30 °C and 0.5 mL/min flow rate. The mobile phases consisted of water (0.1% formic acid, phase A) and acetonitrile (0.1% formic acid, phase B). The elution gradient was as follows: 0–3 min, 5–18% B; 3–10 min, 18–50% B; 10–13 min, 50–90% B; then returned to 5% B for 1 min and re-equilibrated for 2 min.

MS data were acquired with Mass Hunter Workstation software (version B.08.00, Agilent). Ion source conditions were drying gas (N₂) 9 L/min at 280 °C, nebulizer 45 psi, sheath gas 12 L/min at 400 °C, capillary voltage 3500 V, nozzle voltage 500 V, fragmentor 100 V, skimmer 65 V, and octopole RF 750 V. The TOF was set to a scan range of 50–1100 m/z, with a rate of 1.5 spectra/s (666.7 ms/spectrum, 5484 transients/spectrum). Internal mass calibration was performed continuously using reference ions at m/z 112.9855 and 1033.9881 (negative mode) and m/z 121.0509 and 922.0098 (positive mode). Auto-recalibration parameters included a 100 ppm detection window and 1000-count minimum height. For MS/MS analysis, collision energy was set to 20 eV with 100 ms/spectrum acquisition.

All samples were analyzed within a single batch, and injection order was randomized to minimize bias. Quality control (QC) samples, prepared by pooling equal aliquots of each group, were injected at the beginning and end of the run. Methanol blanks were injected every three samples to monitor carry-over.

Feature extraction was performed with Agilent Profinder (version B.06.00) using a pre-filter of ≥ 10,000 counts. Allowed ion species included –H and + HCOO in negative mode and + H in positive mode. Processed features were exported to Mass Profiler Professional 2.0 (Agilent, Santa Clara, CA, USA) for statistical analyses. Unpaired Student’s *t* tests were applied (*p* < 0.05), with Bonferroni–Holm correction for multiple comparisons and a fold-change cutoff of ≥ 2.

Alignment of unidentified features was based on retention time tolerance and spectral similarity. Structural annotation was performed using the MassHunter Molecular Structure Correlator by matching accurate MS/MS fragment ions with candidate structures from databases including PCDL, METLIN, ChemSpider, KEGG, LipidMaps, PubChem, MDB, and FAHFA. Metabolites with scores > 95% were tentatively identified, and when available, validated against authentic standards. Candidate metabolites were prioritized for validation based on both identification score and biological relevance.

#### LCFA analyses by GC–MS

Each intestinal sample (100 mg) was extracted with 1 mL of methyl tert-butyl ether, vortexed for 3 min, and centrifuged at 10,000 rpm for 10 min at 4 °C. The resulting upper organic phase was collected and transferred to vials for analysis. A 1 µL aliquot of each extract was injected into the gas chromatograph.

Long-chain fatty acids (LCFAs) were analyzed with slight modifications to the method of García-Villalba et al. (2012). Chromatographic separation was performed on a VF-WAXms capillary column (30 m × 0.25 mm i.d., 0.25-µm film thickness; Agilent Technologies, Santa Clara, CA, USA) using helium as the carrier gas at a constant flow of 1 mL/min. The injector and MS source temperatures were set to 200 °C and 250 °C, respectively. The oven program was as follows: initial temperature 90 °C; ramp to 150 °C at 15 °C/min; ramp to 170 °C at 5 °C/min; and ramp to 250 °C at 20 °C/min, held for 2 min (total runtime 14 min). The solvent delay was 3.5 min.

Detection was carried out using mass spectrometry with electron impact ionization (EI, 70 eV). The instrument was operated in split mode (split ratio 1:10), with ion source, quadrupole, and transfer line/interface temperatures of 230 °C, 150 °C, and 280 °C, respectively. Mass spectra were recorded in the 50–800 Da range at a scan rate of 2.05 scans/s.

Data acquisition and quantification were performed using *MassHunter* software (Agilent Technologies). Compound identification was achieved by comparing chromatographic retention times and mass spectra with those of authentic standards, published data, and the NIST 2010 GC–MS library. Quantification was based on the summed extracted ion chromatograms (EICs) of diagnostic ions for each analyte.

### Statistical analysis

Because the study aimed to evaluate dietary responses in two biologically distinct intestinal compartments, the statistical analysis considered both diet (control, raw, and hydrolyzed microalgae) and intestinal section (anterior and posterior). Data were analyzed using a factorial statistical model including diet, intestinal section, and their interaction. The objective was to evaluate not only differences among dietary treatments but also whether dietary responses differed between intestinal sections.

For alpha diversity, the normality of the data was first evaluated using the Shapiro–Wilk test for each group. As deviations from normality were detected in some variables and the sample size was limited, non-parametric Kruskal–Wallis tests were applied for all group comparison.

For univariate response variables (relative abundances of major taxa and metabolite concentrations), data were analyzed using two-way analysis of variance (ANOVA) to test the main effects of diet and intestinal section, as well as their interaction (Diet × Intestinal section). When a significant interaction was detected, simple-effects analyses were conducted, followed by pairwise comparisons within each intestinal section, applying appropriate corrections for multiple testing (e.g., Tukey’s HSD).

Given the hierarchical experimental design, tank was considered the experimental unit. Where appropriate, results were additionally verified using linear mixed-effects models, including tank as arandom effect, to account for the non-independence of observations within tanks.

Microbial community composition (beta diversity) was analyzed using permutational multivariate analysis of variance (PERMANOVA) based on Bray–Curtis dissimilarities, including diet, intestinal section, and their interaction as fixed factors. Permutations were constrained to respect the experimental design.

Prior to analysis, data were checked for normality and homogeneity of variances. When necessary, data were transformed to meet model assumptions. Results are presented as mean ± standard error of the mean (SEM), and statistical significance was accepted at *p* < 0.05.

## Results

### Intestinal microbiota

Since both anterior and posterior intestinal sections were analyzed from each fish, the statistical analysis considered both dietary treatment and intestinal compartment in order to evaluate whether dietary responses differed between intestinal sections. Data were initially evaluated using a factorial model including diet, intestinal section (anterior vs posterior), and their interaction. Given the well-established biological and functional differences between anterior and posterior intestinal sections, results are subsequently presented separately for each section. These section-specific analyses represent simple-effects comparisons, with pairwise tests adjusted for multiple comparisons.

#### Alpha and beta diversity

In the factorial analysis of alpha diversity (Shannon index), no significant main effects of diet or intestinal section, nor diet × section interaction, were detected (*p* > 0.05). Therefore, alpha diversity patterns are presented separately for anterior and posterior sections to illustrate section-specific trends.

**Fig. 1:**
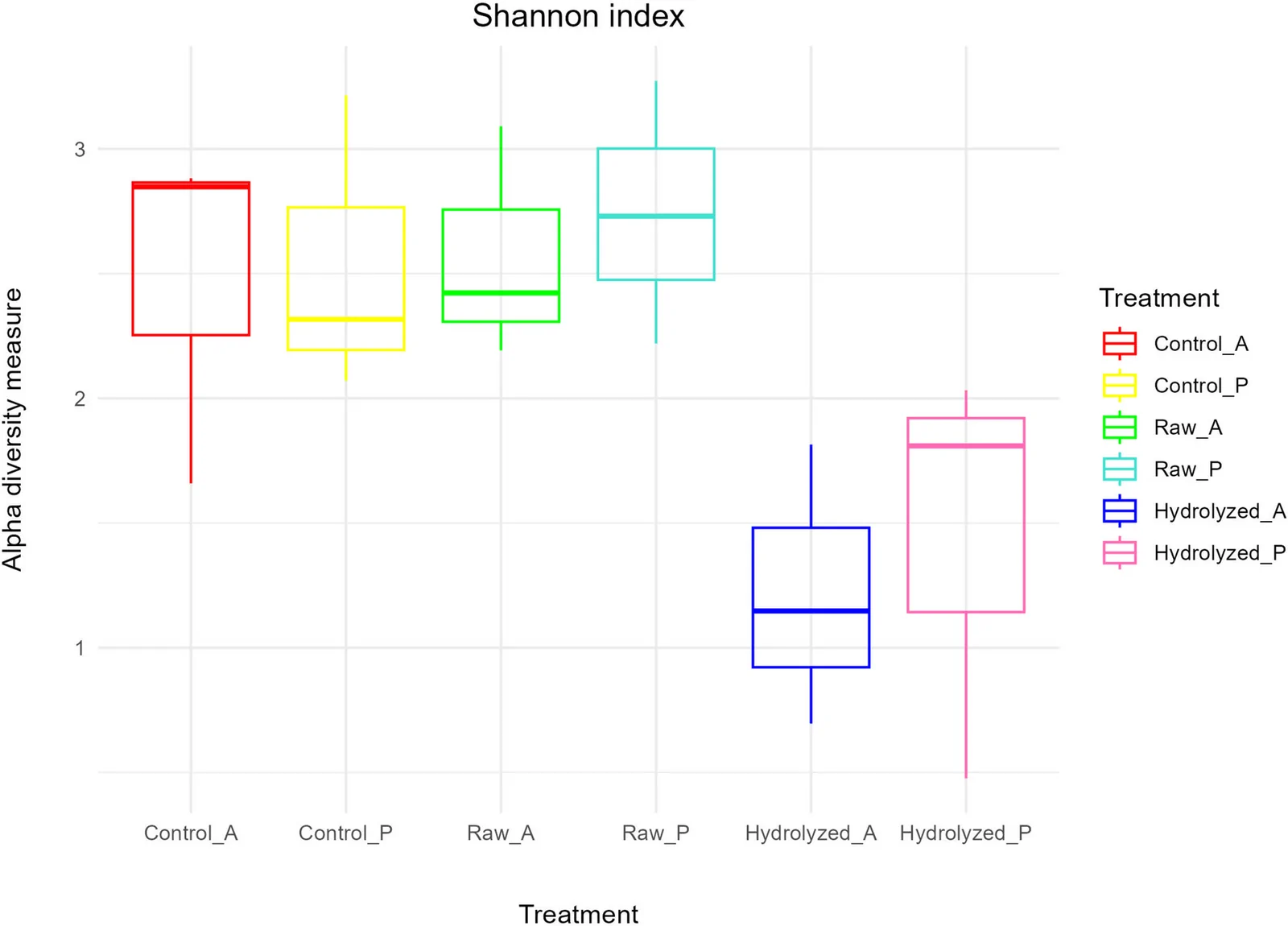
Alpha diversity (Shannon index) of the anterior and posterior intestinal sections of *Sparus aurata* fed with three different diets: control (standard formulation), raw microalgae blend (10%), and hydrolyzed microalgae blend (10%). Values are presented by treatment and intestinal segment (A, anterior; P, posterior). No statistically significant differences were observed among groups

Analysis of alpha diversity, based on the Shannon index, revealed a reduction in microbial diversity in the anterior intestinal section of fish fed the hydrolyzed microalgae diet compared to the control and raw diets. The highest diversity was observed in the control group, followed by the raw diet, with the hydrolyzed diet showing the lowest values. In the posterior section, the raw diet group exhibited the highest alpha diversity, while the hydrolyzed diet remained the lowest. Although these trends were consistent, no statistically significant differences were detected among treatments in either section (Fig. 1). Because some alpha diversity data deviated from normality, non-parametric Kruskal–Wallis tests were applied for group comparisons; however, no statistically significant differences were detected.

**Fig. 2:**
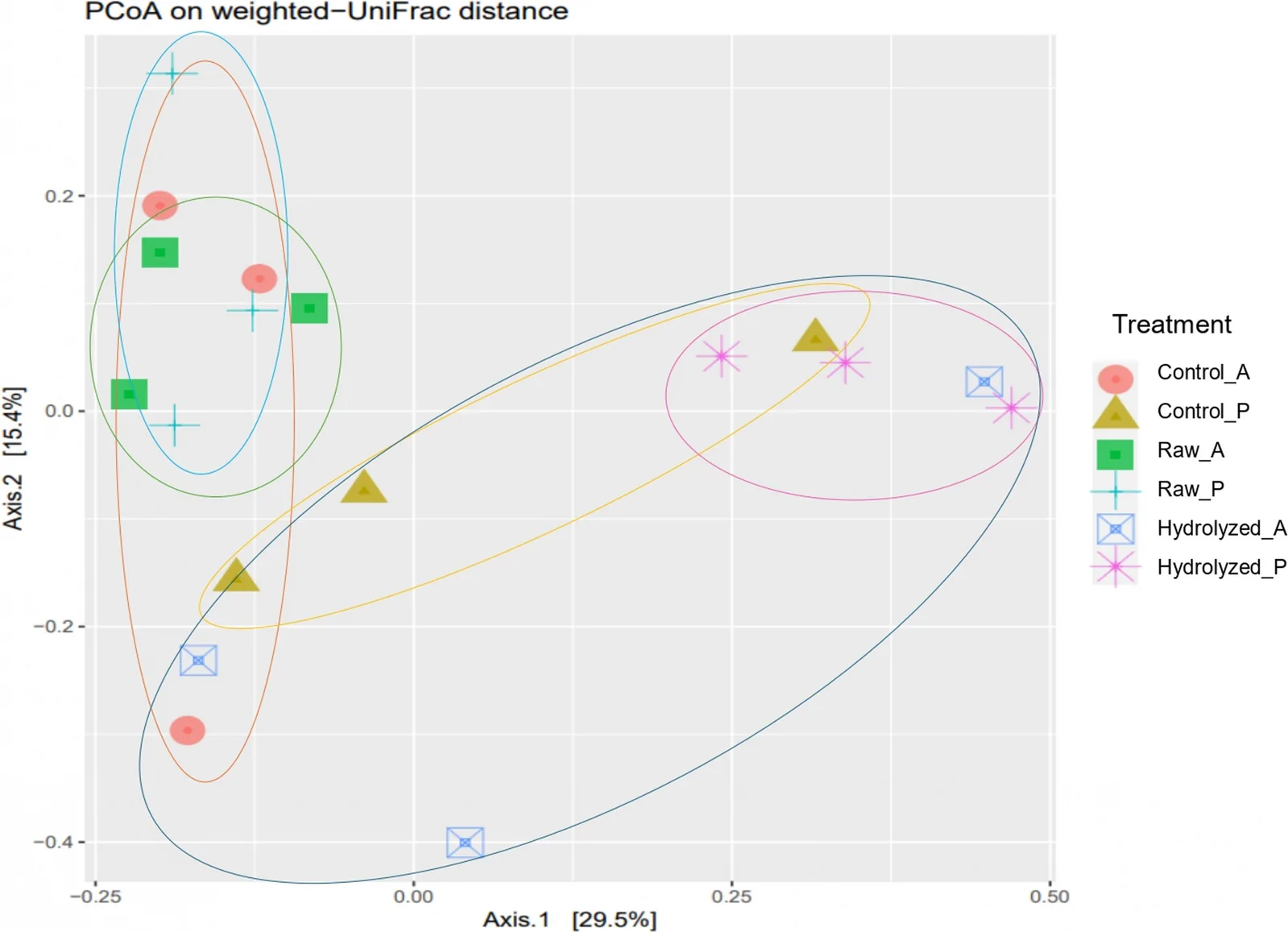
Principal coordinate analysis (PCoA) of beta diversity based on microbial community composition in the anterior (A) and posterior (P) intestinal sections of *Sparus aurata* fed with control, raw microalgae blend (10%), and hydrolyzed microalgae blend (10%) diets

Beta diversity, analyzed through PCoA, demonstrated distinct microbial community structures among dietary treatments. In the anterior intestine, samples from the hydrolyzed group clustered separately from those fed the control or raw diets, indicating a significant shift in community composition. Conversely, in the posterior intestine, the raw diet group formed a separate cluster, while the control and hydrolyzed groups were more similar. PERMANOVA analysis confirmed statistically significant differences in microbial community structure among the three diets (Fig. 2).

PERMANOVA based on Bray–Curtis dissimilarities revealed a significant effect of diet on microbial community composition, as well as a significant effect of intestinal section, while the diet × section interaction was significant (*p* < 0.05). Based on these results, beta diversity patterns were further explored separately for anterior and posterior intestinal sections.

#### Taxonomic composition

Taxonomic differences were interpreted in the context of the factorial analysis described above, with section-specific patterns discussed to reflect biologically meaningful differences between anterior and posterior intestinal compartments.

**Fig. 3:**
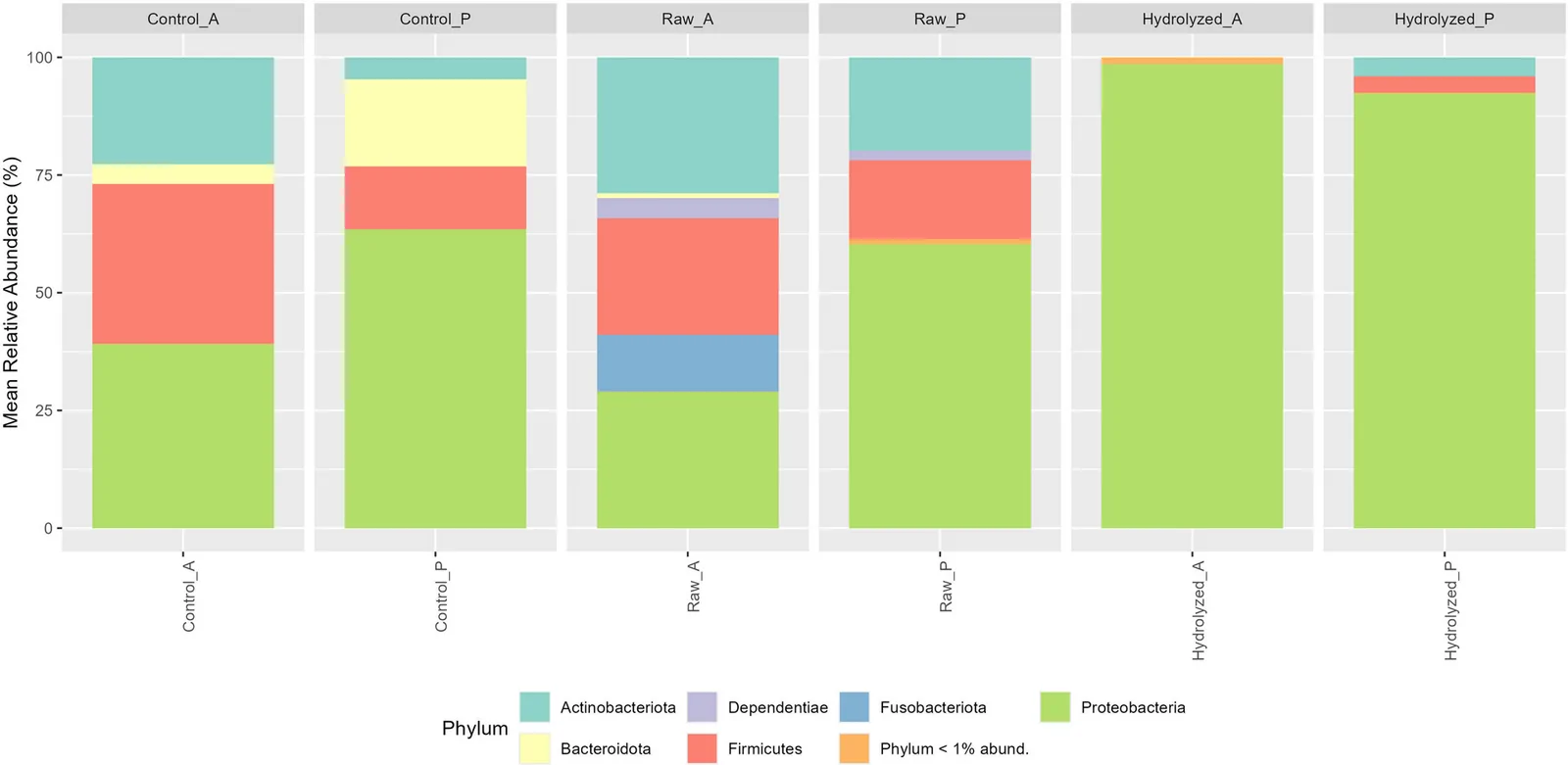
Bacterial phyla found in the sequencing of samples from the anterior (A) and posterior (P) intestinal tracts of specimens of *Sparus aurata* fed with a control diet (control), diet supplemented with raw blend 10% and diet supplemented with hydrolyzed microalgae blend 10%, grouped by treatment and intestinal section

At the phylum level, *Proteobacteria* was the dominant group across all diets and intestinal sections, with relative abundances ranging from 28.94 to 98.54% (Fig. 3). In the control group, *Firmicutes*, *Bacteroidota*, and *Actinobacteria* were also present, contributing to a more balanced microbiota profile. The raw diet promoted a relative increase in *Actinobacteria* and introduced *Fusobacteria* and *Dependientiae*, while reducing *Bacteroidota*. In contrast, the hydrolyzed diet resulted in statistically significant increase in *Proteobacteria* compared to the control (from 39.1 to 98.54%) and raw diet (from 28.94 to 98.54%) in the anterior section, accompanied by a significant sharp decline of *Firmicutes* and complete disappearance of *Bacteroidota* and *Actinobacteria*.

In the posterior intestine, the raw diet increased *Actinobacteria* and reduced *Bacteroidota*, while *Fusobacteria* remained detectable. No significant differences were observed between the control and raw diets, but the hydrolyzed diet again induced a statistically significant increase in *Proteobacteria*.

**Fig. 4:**
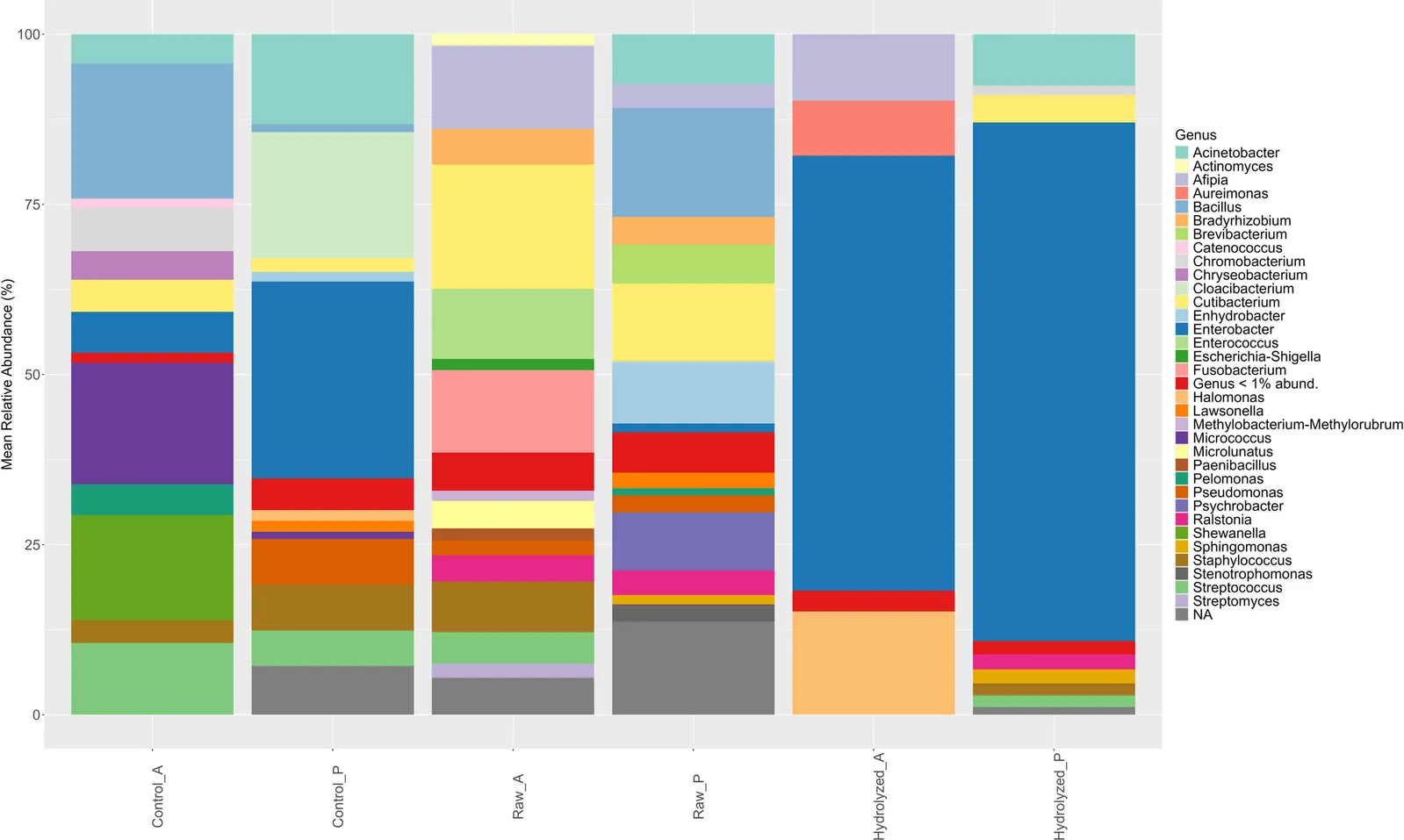
Bacterial genera found in the sequencing of samples from the anterior (A) and posterior (P) intestinal tracts of specimens of *S. aurata* fed with a control diet (control), diet supplemented with raw blend 10% and diet supplemented with hydrolyzed microalgae blend 10%, grouped by treatment and intestinal section

At the genus level (Fig. 4), the control group exhibited a diverse profile with *Bacillus* (19.82%), *Micrococcus* (17.86%), and *Shewanella* (15.44%) as predominant genera in the anterior intestine. In the posterior section, *Enterobacter* (28.91%), *Cloacibacterium* (18.51%), and *Acinetobacter* (13.24%) were most abundant. The raw diet promoted the dominance of *Cutibacterium* (18.56%), *Alfipia* (12.21%), and *Fusobacterium* (10.30%) in the anterior section, and *Bacillus* (15.98%) and *Cutibacterium* (11.44%) in the posterior.

Fish fed the hydrolyzed diet exhibited a highly uneven microbial profile, with *Enterobacter* dominating both intestinal regions (63.92% in the anterior and 76.22% in the posterior section). Other genera such as *Halomonas* (15.19%), *Alfipia* (9.78%), and *Aureimonas* (8.06%) were also detected in the anterior section, while *Acinetobacter* (7.55%) was present in the posterior.

### Intestinal metabolites

Untargeted metabolomics revealed a general decrease in the number of metabolites detected in both microalgae-supplemented diets compared to the control (Table 1). The raw diet retained a higher number of compounds than the hydrolyzed diet, suggesting less disruption of metabolic pathways. Notably, several inflammation-related lipid mediators were consistently reduced in both treatments. These included 9,10-dihydroxy-12,13-epoxyoctadecanoate, 10-hydroperoxy-8E,12Z-octadecadienoic acid, 12-keto-leukotriene B4, 17(R)-resolvin D1, di-n-heptyl phthalate, and 2,3-dinor-11β-PGF2α.

**Table 1:** Decreased list of statistically significant metabolites identified in sea bream gut after different diets

Elemental formula	Tentative metabolite identification	References
Raw diet		
C_18_H_34_O_5_	9,10-Dihydroxy-12,13-epoxyoctadecanoate	METLIN70342
C_18_H_32_O_4_	10-hydroperoxy-8E,12Z-octadecadienoic acid	METLIN35349
C_20_H_30_O_4_	12-Keto-leukotriene B4	HMDB0004234
C_26_H_43_NO_7_S	Sulfolithocholylglycine	HMDB02639
C_22_H_32_O_5_	17(R)-Resolvin D1	Standard
C_20_H_34_O_5_	13,14-dihydro-15-keto-PGF2	HMDB0004685
C_18_H_30_O_4_	(9Z,11R,12S,13S,15Z)−12,13-Epoxy-11-hydroxy-9,15-octadecadienoic acid	HMDB0033505
C_20_H_30_O_5_	15-keto-prostaglandin E2	Standard
C_22_H_34_O_4_	Di-n-heptyl phthalate	HMDB0251285
C_18_H_32_O_5_	Corchorifatty acid F Linoleic acid	HMDB0035919
C_18_H_30_O_5_	2,3-Dinor-11b-PGF2a	Standard
C_26_H_45_NO_7_S	Taurallocholic acid	HMDB0000922
C_18_H_26_O_3_	(9Z,11E,13E,15Z)−4-Oxo-9,11,13,15-octadecatetraenoic acid	HMDB0031098
Hydrolyzed diet		
C_18_H_34_O_5_	9,10-Dihydroxy-12,13-epoxyoctadecanoate	METLIN70342
C_18_H_32_O_4_	10-hydroperoxy-8E,12Z-octadecadienoic acid	METLIN35349
C_20_H_30_O_4_	12-Keto-leukotriene B4	HMDB0004234
C_22_H_32_O_5_	17(R)-Resolvin D1	Standard
C_20_H_34_O_5_	13,14-dihydro-15-keto-PGF2	HMDB0004685
C_20_H_30_O_5_	15-keto-prostaglandin E2	Standard
C_22_H_34_O_4_	Di-n-heptyl phthalate	HMDB0251285
C_18_H_30_O_5_	2,3-Dinor-11b-PGF2a	Standard
C_18_H_37_O_9_P	PG (12:0/0:0)	METLIN79999

These findings point toward a potential anti-inflammatory effect of both microalgae-based diets, particularly through the suppression of key eicosanoids and prostaglandin derivatives. The hydrolyzed diet, despite its lower metabolite richness, showed a more pronounced reduction in compounds associated with inflammatory signaling.

Long-chain fatty acid (LCFA) profiles, analyzed by GC–MS (Table 2), showed higher overall abundance in the posterior intestinal sections across all diets. In the anterior sections, the composition varied depending on the diet, with limited consistency among groups.

**Table 2:** List of long-chain fatty acids present in the anterior and posterior sections of the gut of fish feed with the different diets

		Elemental formula	Tentative metabolite identification	References
Control diet	**Anterior part**	C₁₄H₂₈O₂	Tetradecanoic acid (TR 29,9)	HMDB0000806
	**Posterior part**	C₁₄H₂₈O₂	Tetradecanoic acid (TR 29,9)	HMDB0000806
		C₁₅H₃₀O₂	Pentadecanoic acid (TR 31,0)	HMDB0000826
		C₁₆H₃₂O₂	n-hexadecanoic acid (TR 32,2)	HMDB0000220
		C₁₆H₃₀O₂	Palmitoleic acid (TR 32,7)	HMDB0003229
Raw diet	**Anterior part**	C₁₆H₃₂O₂	n-hexadecanoic acid (TR 32,2)	HMDB0000220
	**Posterior part**	C₁₄H₂₈O₂	Tetradecanoic acid (TR 29,9)	HMDB0000806
		C₁₆H₃₂O₂	n-hexadecanoic acid (TR 32,2)	HMDB0000220
		C₁₆H₃₀O₂	Palmitoleic acid (TR 32,7)	HMDB0003229
		C₁₈H₃₆O₂	Octadecanoic acid (TR 35,3)	HMDB0000827
		C₁₈H₃₄O₂	Oleic acid (TR 35,9)	HMDB0000207
Hydrolized diet	**Anterior part**	C₁₅H₃₀O₂	Pentadecanoic acid (TR 31,0)	HMDB0000826
		C₁₈H₃₄O₂	Octadecenoic acid (TR 36,0)	HMDB0245277
	**Posterior part**	C₁₄H₂₈O₂	Tetradecanoic acid (TR 29,9)	HMDB0000806
		C₁₅H₃₀O₂	Pentadecanoic acid (TR 31,0)	HMDB0000826
		C₁₆H₃₀O₂	Palmitoleic acid (TR 32,7)	HMDB0003229
		C₁₈H₃₆O₂	Octadecanoic acid (TR 35,3)	HMDB0000827
		C₁₈H₃₄O₂	Oleic acid (TR 35,9)	HMDB0000207

In the posterior intestine, tetradecanoic and palmitoleic acids were consistently present in all treatment groups, indicating their conserved role in intestinal lipid metabolism. Octadecanoic (stearic) and oleic acids appeared exclusively in fish fed microalgae-containing diets, both raw and hydrolyzed, highlighting an improvement in the intestinal absorption or availability of these beneficial fatty acids. These compounds are associated with improved feed efficiency, lipid regulation, and anti-inflammatory activity, suggesting functional benefits of microalgae supplementation.

## Discussion

This study evaluated the impact of dietary supplementation with a blend of *M. gaditana* and *A. platensis*, either raw or enzymatically hydrolyzed, on the intestinal microbiota and metabolomic profile of *S. aurata*. The results indicate that the processing method of the microalgal biomass plays a key role in shaping both gut microbial communities and intestinal metabolic outputs, highlighting the importance of integrating microbial and metabolomic responses to fully interpret the biological implications of dietary interventions.

Alpha diversity analysis revealed a clear decrease in microbial diversity in fish fed the hydrolyzed microalgae diet, consistent with previous observations that enzymatic treatments may alter microbial colonization patterns by enhancing the bioavailability of low molecular weight compounds (Bongiorno et al. 2022). Although reduced diversity is sometimes associated with compromised gut resilience (Kormas et al. 2014), it may also reflect a shift toward a more functionally specialized community, improving nutrient utilization (Estruch et al. 2015). The raw microalgae diet, on the other hand, maintained microbial diversity similar to the control group, suggesting a less disruptive effect on microbial ecology but potentially lower digestibility and metabolic activation, as indicated in earlier growth studies (Molina-Roque et al. 2022).

Beta diversity analyses supported these findings, with the hydrolyzed group displaying distinct clustering, especially in the anterior intestinal section, confirming that enzymatic hydrolysis altered the microbial composition. Similar effects have been reported with hydrolyzed microalgae in other aquaculture species (Galafat et al. 2020; 2022), where processed biomass induced changes in mucosal integrity and enzymatic activity. These findings underscore the importance of biomass processing in modulating gut microbiota structure. These findings are in line with previous work by Molina-Roque et al. (2022), who demonstrated that the inclusion of enzymatically hydrolyzed microalgal products improved growth performance, intestinal permeability, and hepatic carbohydrate metabolism in *Sparus aurata* close to commercial size. Their study also highlighted those biotechnological treatments enhanced the intestinal absorptive capacity and nutrient assimilation efficiency, mechanisms that may underline the microbiota and metabolomic shifts observed in our work.

At the phylum level, *Proteobacteria* was dominant across all treatments, a common feature in marine fish microbiota (Kormas et al. 2014). However, its extreme abundance in the hydrolyzed diet (up to 98.54%) may signal dysbiosis, as excessive *Proteobacteria* have been associated with inflammatory states and metabolic stress in fish (Shin et al. 2015). The concurrent reduction of *Firmicutes* and *Bacteroidota* in the hydrolyzed group, both known for their role in fiber degradation and production of beneficial short-chain fatty acids (Estruch et al. 2015), suggests a functional imbalance.

In contrast, the raw diet preserved higher levels of *Actinobacteria* and *Firmicutes* and uniquely induced the appearance of *Fusobacteria* and *Dependientiae*. *Fusobacteria* has been proposed as a marker of intestinal health in cyprinid fish (Luan et al. 2023), and its presence may indicate beneficial fermentative activity. The application of the ratio [Fusobacteria + Firmicutes + Bacteroidota)/Proteobacteria] proposed by Luan et al. (2023) further supports that the raw diet promotes a more balanced microbiota, especially in the anterior section. This contrasts with the hydrolyzed group, in which this ratio dropped sharply, suggesting compromised gut microbial balance.

At the genus level, distinct patterns emerged. In the control group, *Bacillus*, *Micrococcus*, and *Shewanella* were dominant, all genera with recognized probiotic or metabolic benefits. *Shewanella* species, in particular, produce omega-3 fatty acids and support lipid metabolism (Tapia-Paniagua et al. 2014; Lozano-Muñoz et al. 2020), while *Bacillus* strains are widely used as probiotics in aquaculture (Rimoldi et al. 2020). Their disappearance in the raw and hydrolyzed diets require further investigation. Notably, the hydrolyzed group showed a heavy dominance of *Enterobacter*, a genus that includes both probiotic and opportunistic species (LaPatra et al. 2014; Amaro et al. 2008). The loss of microbial evenness and emergence of potential pathobionts highlight the need for caution when using hydrolyzed products.

The metabolomic profiles differed markedly between treatments, providing additional insight into the functional consequences of dietary modulation. Both microalgae-based diets were associated with reduced levels of inflammation-related metabolites, including 12-keto-leukotriene B4 and 15-keto-prostaglandin E2, compounds involved in eicosanoid signaling and immune regulation in fish (Ni and Liu 2021; Alba et al. 2023). These results suggest a modulation of inflammation-related metabolic pathways, potentially associated with dietary microalgae inclusion and increased PUFA availability, as previously reported for microalgal diets (Sáez et al. 2022; Mueller et al. 2023). Nevertheless, the absence of classical inflammatory markers limits definitive conclusions regarding anti-inflammatory effects.

The detection of beneficial long-chain fatty acids, such as oleic and stearic acids, exclusively in microalgae-fed groups, further supports improved lipid metabolism and nutrient assimilation. Oleic acid has been associated with enhanced feed efficiency and reduced oxidative stress (Martins et al. 2024;Santa-María et al. 2023), whereas stearic acid plays a role in hepatic lipid homeostasis (Martins et al. 2024). In addition, conserved detection of tetradecanoic and palmitoleic acids in the posterior intestine across all treatments suggests a stable role in host–microbiota interactions (Weimann et al. 2018; Albaladejo-Riad et al. 2024). Variations in bile acid-related compounds, including taurallocholic acid and sulfolithocholylglycine, further indicate that dietary microalgae may influence bile acid metabolism, potentially enhancing lipid digestion and reducing enterohepatic recycling (Collins et al. 2023).

Several methodological considerations should be acknowledged when interpreting these results. The focus on anterior and posterior intestinal sections captures major functional contrasts along the gut, although finer spatial resolution across the entire intestinal tract could provide additional insight into whole-gut microbial dynamics. Moreover, the standardized overnight fasting applied prior to sampling, while reducing postprandial variability and digesta-associated noise, may have influenced microbial activity. Additionally, although 16S rRNA gene amplicon sequencing provides robust information on bacterial community structure, it does not capture other intestinal microorganisms or functional gene content, highlighting the need for integrative multi-omics approaches in future studies.

Taken together, the observed microbial and metabolomic responses reveal a trade-off between intestinal microbial stability and metabolic responsiveness depending on the processing of the microalgal biomass. The raw microalgae blend preserved bacterial diversity and promoted a more balanced microbial community, which may be advantageous for maintaining gut homeostasis. In contrast, the hydrolyzed blend, characterized by increased nutrient and PUFA bioavailability, induced stronger metabolic and microbial shifts, consistent with enhanced physiological responsiveness but potentially requiring closer monitoring of intestinal balance.

From an applied perspective, these findings support the strategic use of *M. gaditana* and *A. platensis* blends as functional ingredients in gilthead seabream finishing diets. Raw microalgae may be preferentially applied when the goal is to preserve gut microbial stability, whereas enzymatically hydrolyzed microalgae could be used to enhance growth performance, metabolic activity, and fillet quality, as previously reported. This flexible, goal-oriented feeding strategy may allow producers to maximize functional benefits while minimizing potential risks, reinforcing the relevance of tailored dietary interventions in sustainable aquaculture.

The present study complements previously published data from the same feeding trial (Molina-Roque et al. 2022), which demonstrated significant improvements in growth performance and welfare indicators, providing a mechanistic framework linking dietary microalgae supplementation to intestinal microbial and metabolic modulation.

In summary, this study provides evidence that dietary inclusion of raw and hydrolyzed *M. gaditana* and *A. platensis* can modulate the intestinal microbiota and metabolomic composition of *S. aurata*. The hydrolyzed form exhibits stronger metabolic modulation but may disrupt microbial balance, whereas the raw form maintains microbial diversity and supports gut health markers. Future research should focus on optimizing these processes, conducting long-term trials, and performing integrative microbiome-metabolome analyses to validate these findings and ensure the safety and efficacy of algae-based aquafeed strategies.

## Conclusions

This study demonstrates that dietary supplementation with a *M. gaditana* and *A. platensis* blend, especially in its hydrolyzed form, modulates the intestinal microbiota and metabolic profile of *S. aurata*. While the hydrolyzed diet improved the presence of anti-inflammatory mediators and beneficial long-chain fatty acids, it also caused a significant loss in microbial diversity and an excessive proliferation of Proteobacteria, which may reflect a dysbiotic state.

In contrast, the raw biomass preserved microbial diversity and fostered the presence of fermentative phyla like *Fusobacteria*, suggesting improved gut health potential. The exclusive detection of stearic and oleic acids in both algae-supplemented diets highlights the functional metabolic benefits of microalgae inclusion.

These findings support the use of microalgae as sustainable feed additives capable of partially replacing fishmeal while promoting metabolic resilience. However, future studies should address the optimal dosage and processing conditions, and apply integrative multi-omics approaches to clarify the functional links between microbiota changes and metabolomic shifts. Long-term trials under commercial conditions are also recommended to ensure efficacy and safety in aquaculture systems.

## Supplementary Information

Below is the link to the electronic supplementary material.

ESM 1 (DOCX 20.0 KB)

ESM 2 (DOCX 28.6 KB)

## Data Availability

The raw sequence data generated for the intestinal microbiota analysis have been deposited in the NCBI Sequence Read Archive (SRA) under accession number PRJNA1268883 and are publicly available.
