# Supplementary material for: The diet supplemented with raw and hydrolyzed Microchloropsis gaditana and Arthrospira platensis blend modulates intestinal microbiota and metabolomic profiles in Sparus aurata
Source: Fish Physiol Biochem. 2026 Sep 14;52(5):168. doi: 10.1007/s10695-026-01791-0 (PMC13574860; doi:10.1007/s10695-026-01791-0)
Supplement: Supplementary file 1 [file 10695_2026_1791_MOESM1_ESM.docx]

| **Rt (min)** | ***m/z* experimental** | ***m/z* theoretical** | **Ion annotation** | **Fragments** | **Elemental formula*^a^*** | **Tentative metabolite identification** | **Diets** | **References** |
| --- | --- | --- | --- | --- | --- | --- | --- | --- |
| 18.42 | 329.2325 | 329.2333 | [M–H]^-^ |  | C_18_H_34_O_5_ | 9,10-Dihydroxy-12,13-epoxyoctadecanoate | Raw | METLIN70342 |
| 23.06 | 311.2220 | 311.2228 | [M–H]^-^ |  | C_18_H_32_O_4_ | 10-hydroperoxy-8E,12Z-octadecadienoic acid | Raw | METLIN35349 |
| 22.37 | 333.2064 | 333.2071 | [M–H]^-^ | 315.1966 | C_20_H_30_O_4_ | 12-Keto-leukotriene B4 | Raw | HMDB0004234 |
| 16.42 | 512.2680 | 512.2687 | [M–H]^-^ | 468.2789 | C_26_H_43_NO_7_S | Sulfolithocholylglycine | Raw | HMDB02639 |
| 20.11 | 375.2170 | 375.2177 | [M–H]^-^ |  | C_22_H_32_O_5_ | 17(R)-Resolvin D1 | Raw | Standard |
| 19.47 | 353.2326 | 353.2333 | [M–H]^-^ | 335.2228 | C_20_H_34_O_5_ | 13,14-dihydro-15-keto-PGF2 | Raw | HMDB0004685 |
| 21.91 | 309.2062 | 309.2071 | [M–H]^-^ | 265.2168 | C_18_H_30_O_4_ | (9Z,11R,12S,13S,15Z)-12,13-Epoxy-11-hydroxy-9,15-octadecadienoic acid | Raw | HMDB0033505 |
| 18.78 | 349.2018 | 349.2020 | [M–H]^-^ |  | C_20_H_30_O_5_ | 15-keto-Prostaglandin E2 | Raw | Standard |
| 22.00 | 361.2375 | 361.2384 | [M–H]^-^ | 219.1390 | C_22_H_34_O_4_ | Di-n-heptyl phthalate | Raw | HMDB0251285 |
| 17.72 | 327.2170 | 327.2177 | [M–H]^-^ | 211.1345 | C_18_H_32_O_5_ | Corchorifatty acid F  Linoleic acid | Raw | HMDB0035919 |
| 17.36 | 325.2016 | 325.2020 | [M–H]^-^ |  | C_18_H_30_O_5_ | 2,3-Dinor-11b-PGF2a  (Mastocite content) | Raw | Standard |
| 15.40 | 512.2680 | 512.2687 | [M–H]^-^ | 496.2738 | C_26_H_45_NO_7_S | Taurallocholic acid  (sales biliares) | Raw | HMDB0000922 |
| 21.37 | 289.1902 | 289.1909 | [M–H]^-^ | 271.1703 | C_18_H_26_O_3_ | (9Z,11E,13E,15Z)-4-Oxo-9,11,13,15-octadecatetraenoic acid | Raw | HMDB0031098 |
| 19.48 | 329.2325 | 329.2333 | [M–H]^-^ |  | C_18_H_34_O_5_ | 9,10-Dihydroxy-12,13-epoxyoctadecanoate | Hidrolized | METLIN70342 |
| 22.73 | 311.2220 | 311.2228 | [M–H]^-^ |  | C_18_H_32_O_4_ | 10-hydroperoxy-8E,12Z-octadecadienoic acid | Hidrolized | METLIN35349 |
| 22.37 | 333.2064 | 333.2071 | [M–H]^-^ | 315.1966 | C_20_H_30_O_4_ | 12-Keto-leukotriene B4 | Hidrolized | HMDB0004234 |
| 20.11 | 375.2170 | 375.2177 | [M–H]^-^ |  | C_22_H_32_O_5_ | 17(R)-Resolvin D1 | Hidrolized | Standard |
| 19.47 | 353.2326 | 353.2333 | [M–H]^-^ | 335.2228 | C_20_H_34_O_5_ | 13,14-dihydro-15-keto-PGF2 | Hidrolized | HMDB0004685 |
| 18.78 | 349.2018 | 349.2020 | [M–H]^-^ |  | C_20_H_30_O_5_ | 15-keto-Prostaglandin E2 | Hidrolized | Standard |
| 22.00 | 361.2375 | 361.2384 | [M–H]^-^ | 219.1390 | C_22_H_34_O_4_ | Di-n-heptyl phthalate | Hidrolized | HMDB0251285 |
| 17.36 | 325.2016 | 325.2020 | [M–H]^-^ |  | C_18_H_30_O_5_ | 2,3-Dinor-11b-PGF2a | Hidrolized | Standard |
| 23.54 | 427.2100 | 427.2102 | [M–H]^-^ |  | C_18_H_37_O_9_P | PG(12:0/0:0) | Hidrolized | METLIN79999 |
