# Supplementary material for: The diet supplemented with raw and hydrolyzed Microchloropsis gaditana and Arthrospira platensis blend modulates intestinal microbiota and metabolomic profiles in Sparus aurata
Source: Fish Physiol Biochem. 2026 Sep 14;52(5):168. doi: 10.1007/s10695-026-01791-0 (PMC13574860; doi:10.1007/s10695-026-01791-0)
Supplement: Supplementary file 2 [file 10695_2026_1791_MOESM2_ESM.docx]

**Supplementary table S1.** Ingredient composition of the experimental diets.

|  | **Diets** | | |
| --- | --- | --- | --- |
| ***Ingredient composition***  (g 100 g-1 dry matter) | **Control** | **Raw** | **Hydrolyzed** |
| Fish meal LT94^1^ | 15.0 | 15.0 | 15.0 |
| Lysine^2^ | 1.2 | 1.2 | 1.2 |
| Methionine^2^ | 0.5 | 0.5 | 0.5 |
| Squid meal^3^ | 1.0 | 1.0 | 1.0 |
| Fish meal hydrolysate^4^ | 0.5 | 0.5 | 0.5 |
| *LB-ChromaBream*^5^ | - | 10 | - |
| *LB-ChromaBream-plus*^5^ | - | - | 10 |
| Wheat gluten^6^ | 15.0 | 13.0 | 13.0 |
| Soybean protein concentrate^7^ | 35.0 | 33.0 | 33.0 |
| Fish oil^8^ | 5.0 | 4.5 | 4.5 |
| Soybean oil^9^ | 8.0 | 7.2 | 7.2 |
| Soybean lecithin^10^ | 1.0 | 1.0 | 1.0 |
| Wheat meal^11^ | 12.7 | 8.0 | 8.0 |
| Choline chloride^12^ | 0.5 | 0.5 | 0.5 |
| Betain^13^ | 0.5 | 0.5 | 0.5 |
| Vitamin and mineral premix^14^ | 2.1 | 2.1 | 2.1 |
| Vitamin C^15^ | 0.1 | 0.1 | 0.1 |
| Guar gum^16^ | 2.0 | 2.0 | 2.0 |
| Crude protein | 42.5 ± 0.8 | 42.1 ± 1.2 | 43.0 ± 1.7 |
| Crude lipid | 14.2 ± 0.3 | 14.5± 0.2 | 13.8 ± 0.9 |
| Ash | 6.0 ± 0.5 | 5.8 ± 0.1 | 5.9 ± 0.0 |

Dietary treatments: control diet; **Raw:** diet including 100 g kg^-1^ *LB-Chromabream* additive; **Hydrolyzed:** diet including 100 g kg^-1^ *LB-ChromabreamPlus* additive.

^1^(protein: BA.4 %; lipid: 12.G %), Norsildemel (Bergen, Norway); ^2^ Suysegala (Sevilla, Spain); ^3^Bacarel (UL); ^4^ CPSP (Sopropeche, France); ^5^ Lifebioencapsulation SL (Almería, Spain); ^6^ (protein: 7B.0 %; lipid: 1.A %), Lorca Nutricion Animal (Murcia, Spain); ^7^ (protein: 50.0 %; lipid: 1.0 %) Lorca Nutricion Animal (Murcia, Spain); ^8^ AFAMPES 117ECA (AFAMSA, Spain);^9^Aceites el Niño (Málaga, Spain); ^10^Lecico P700 (Lecico GmbC, EE); ^11^(protein: 12.0 %; lipid: 2.0 %), local provider; ^12,13^ Sigma-Aldrich (Madrid, Spain); ^14,15^ Vitamin & Mineral Premix: Vitamins (IU or mg kg−1 premix): vitamin A (retinyl acetate), 2000,000 IU; vitamin EG (EL-cholecalciferol), 200,000 IU; vitamin E, 10,000 mg; vitamin LG (menadione sodium bisulphite), 2500 mg; vitamin B1(thiamine hydrochloride), G000 mg; vitamin B2 (riboKavin), G000 mg; calcium pantothenate, 10,000 mg; nicotinic acid, 20,000 mg; vitamin BB (pyridoxine hydrochloride), 2000 mg; vitamin BA (folic acid), 1500 mg; vitamin B12 (cyanocobalamin), 10 mg vitamin C (biotin), G00 mg; inositol, 50,000 mg; betaine, 50,000 mg; vitamin C (ascorbic acid), 50,000 mg. Minerals (mg kg−1 premix): Co (cobalt carbonate), B5 mg; Cu (cupric sulphate), A00 mg; Fe (iron sulphate), B00 mg; I (potassium iodide), 50 mg; Mn (manganese oxide), AB0 mg; Se (sodium selenite), 1 mg; Nn (zinc sulphate) 750 mg; Ca (calcium carbonate), 18B,000 mg; LCl, 24,100 mg; NaCl 40,000 mg; excipient sepiolite, colloidal silica (LifeBioencapsulation premix); ^16^ EPSA (Sevilla, Spain).

**Supplementary table S2.** Fatty acid profile of the experimental diets (% of total fatty acids).

|  |  | **Experimental diets** | | |  |
| --- | --- | --- | --- | --- | --- |
|  |  | **Control** | **Raw** | **Hydrolyzed** | ***p*** |
| 14:0 |  | 1.43±0.01^a^ | 1.62±0.01^b^ | 1.58±0.02^b^ | *0.001* |
| 16:0 |  | 14.09±0.05^a^ | 14.79±0.06^b^ | 14.66±0.08^b^ | *0.003* |
| 18:00 |  | 4.44±0.04^b^ | 4.3±0.01^a^ | 4.35±0.02^ab^ | *0.027* |
| 16:1n-7 |  | 2.14±0.04 | 1.76±0.67 | 2.26±0.02 | *n.s.* |
| 18:1n-7 |  | 1.23±0.04 | 1.25±0.01 | 1.26±0.01 | *n.s.* |
| 18:1n-9 |  | 21.03±0.06^b^ | 20.42±0.09^a^ | 20.5±0.06^a^ | *0.006* |
| 20:1n-9 |  | 0.05±0.07 | 0.05±0.07 | 0.05±0.07 | *n.s.* |
| 18:2n-6 |  | 37.86±0.12^b^ | 36.53±0.09^a^ | 36.57±0.26^a^ | *0.008* |
| 18:3n-3 |  | 0.96±0.14a | 1.32±0.01b | 1.24±0.00ab | *0.043* |
| 16:2n4 |  | 0.29±0.06 | 0.3±0.06 | 0.29±0.06 | *n.s.* |
| 16:3n4 |  | 0.44±0.06 | 0.44±0.06 | 0.44±0.06 | *n.s.* |
| 18:4n-3 |  | 0.86±0.02 | 0.82±0.01 | 0.85±0.01 | *n.s.* |
| 20:4n-6 |  | 0.73±0.22 | 0.56±0.01 | 0.57±0.02 | *n.s.* |
| 20:4n-3 |  | 0.15±0.01 | 0.14±0.02 | 0.14±0.01 | *n.s.* |
| 20:5n-3 (EPA^1^) |  | 2.99±0.01 | 2.98±0.01 | 2.85±0.20 | *n.s.* |
| 22:5n-3 |  | 0.38±0.01 | 0.38±0.01 | 0.38±0.01 | *n.s.* |
| 22:6n-3 (DHA^2^) |  | 7.49±0.12 | 7.37±0.03 | 7.44±0.07 | *n.s.* |
| ∑SFAs^3^ |  | 19.96±0.02^a^ | 20.71±0.07^b^ | 20.59±0.07^b^ | *0.002* |
| ∑MUFAs^4^ |  | 24.45±0.01 | 23.48±0.64 | 24.07±0.02 | *n.s.* |
| ∑PUFAs^5^ |  | 56.3±0.09^b^ | 54.84±0.09^a^ | 54.84±0.53^a^ | *0.029* |
| ∑PUFAs n-3 |  | 12.83±0.27 | 13.02±0.06 | 12.91±0.27 | *n.s.* |
| ∑PUFAs n-6 |  | 38.6±0.34^b^ | 37.08±0.1^a^ | 37.15±0.29^a^ | *n.s.* |
| n-3/n-6 |  | 0.33±0.01 | 0.35±0.00 | 0.35±0.00 | *n.s.* |
| EPA/DHA |  | 0.40±0.01 | 0.40±0.00 | 0.38±0.02 | *n.s.* |
| PUFA/SFA |  | 2.82±0.01^b^ | 2.64±0.01^a^ | 2.66±0.01^a^ | *0.006* |

Dietary treatments: Raw diet including 100 g kg^-1^ *LB-Chromabream* additive; Hydrolyzed diet including 100 g kg^-1^ *LB-ChromabreamPlus* additive. Values with different lowercase superscript indicate significant differences attributed to dietary treatments (P<0.05). ^1^EPA: eicosapentaenoic acid; ^2^DHA: docosahexaenoic acid. ^3^SFAs: saturated fatty acids; ^4^MUFAs: monounsaturated fatty acids; ^5^PUFAs: polyunsaturated fatty acids. Values are expressed as average ± sd (n=3). *n.s*.: not significant.

**Supplementary Table S3.**
Summary of growth performance, somatic indices, and stress markers previously reported in Molina-Roque et al. (2022) for the same experimental trial. Dietary treatments were control diet; Raw**:** diet including 100 g kg^-1^ *LB-Chromabream* additive; Hydrolyzed: diet including 100 g kg^-1^ *LB-ChromabreamPlus* additive.

|  | **control** | **Raw** | **Hydrolyzed** | [pa](https://www.sciencedirect.com/science/article/pii/S2352513422002447?via%3Dihub#tbl2fna) |  |
| --- | --- | --- | --- | --- | --- |
| **Initial body mass (g)** | 182,50 ± 0,16 | 182,60 ± 0,30 | 182,60 ± 0,17 | 0.956 |  |
| **Final body mass (g)** | 233,0 ± 0,66^a^ | 240,8 ± 3,20^ab^ | 247,7 ± 2,42^b^ | 0.013 |  |
| [Kb](https://www.sciencedirect.com/science/article/pii/S2352513422002447?via%3Dihub#tbl2fnb) | 2,06 ± 0,03 | 2.00 ± 0.02 | 2,01 ± 0,03 | 0.169 |  |
| [WG (%)](https://www.sciencedirect.com/science/article/pii/S2352513422002447?via%3Dihub#tbl2fnc)^[c](https://www.sciencedirect.com/science/article/pii/S2352513422002447?via%3Dihub#tbl2fnc)^ | 27,65 ± 0,47^a^ | 31,89 ± 1,54^ab^ | 35.63 ± 1.20^b^ | 0.008 |  |
| [SGR (%)](https://www.sciencedirect.com/science/article/pii/S2352513422002447?via%3Dihub#tbl2fnd)^[d](https://www.sciencedirect.com/science/article/pii/S2352513422002447?via%3Dihub#tbl2fnd)^ | 0,59 ± 0,01^a^ | 0,67 ± 0,03^ab^ | 0,74 ± 0,02^b^ | 0.008 |  |
| [FIe](https://www.sciencedirect.com/science/article/pii/S2352513422002447?via%3Dihub#tbl2fne) | 78,65 ± 0,89a | 80,48 ± 1,44^a^ | 87,06 ± 0,52^b^ | 0.003 |  |
| [FEf](https://www.sciencedirect.com/science/article/pii/S2352513422002447?via%3Dihub#tbl2fnf) | 0,64 ± 0,02^a^ | 0,72 ± 0,03^ab^ | 0,75 ± 0,02^b^ | 0.047 |  |
| [HSI (%)](https://www.sciencedirect.com/science/article/pii/S2352513422002447?via%3Dihub#tbl2fng)^[g](https://www.sciencedirect.com/science/article/pii/S2352513422002447?via%3Dihub#tbl2fng)^ | 1,32 ± 0,05 | 1.21 ± 0.04 | 1,25 ± 0,08 | 0.445 |  |
| [MSI (%)](https://www.sciencedirect.com/science/article/pii/S2352513422002447?via%3Dihub#tbl2fnh)^[h](https://www.sciencedirect.com/science/article/pii/S2352513422002447?via%3Dihub#tbl2fnh)^ | 1.15 ± 0.17 | 1,57 ± 0,22 | 1.24 ± 0.11 | 0.216 |  |
| [ILI (%)](https://www.sciencedirect.com/science/article/pii/S2352513422002447?via%3Dihub#tbl2fni)^[i](https://www.sciencedirect.com/science/article/pii/S2352513422002447?via%3Dihub#tbl2fni)^ | 74.07 ± 3.15^a^ | 88.44 ± 3.18^b^ | 85,70 ± 2,35^b^ | 0.003 |  |
|  |  |  |  |  |  |

^a^ Values resulting from one-way analysis of variance.

b Condition factor: (100 × body weight)/fork length3.

c Weight gain (%): (100 × body weigh increase)/initial body weight.

d Specific growth rate: 100 × (ln final body weight ln initial body weight)/ days.

e Feed Intake: grams of aquafeed consumed/tank)/week.

f Feed Efficiency: weight gain/total feed intake.

g Hepatosomatic index: (100 × liver weight)/fish weight.

h Mesenteric index: (100 × perivisceral fat weight)/fish weight.

i Intestine length index: (100 × intestine length)/fork length.
